# Rational Construction of Two-Dimensional Conjugated Metal–Organic Frameworks (2D c-MOFs) for Electronics and Beyond

**DOI:** 10.1021/acs.accounts.4c00305

**Published:** 2024-07-04

**Authors:** Yang Lu, Paolo Samorì, Xinliang Feng

**Affiliations:** † Université de Strasbourg, CNRS, ISIS, UMR 7006, 8 Alleé Gaspard Monge, 67000 Strasbourg, France; ‡ Max Planck Institute of Microstructure Physics, 06120 Halle (Saale), Germany; § Center for Advancing Electronics Dresden and Faculty of Chemistry and Food Chemistry, Technische Universität Dresden, 01067 Dresden, Germany

## Abstract

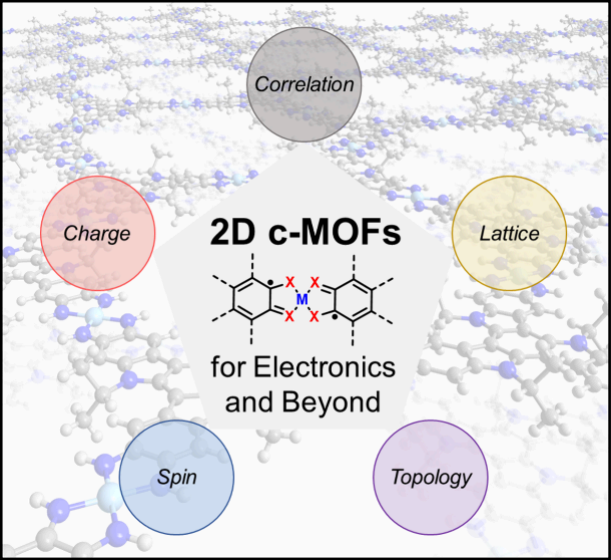

Two-dimensional conjugated metal–organic frameworks (2D c-MOFs) have emerged as a novel class of multifunctional materials, attracting increasing attention due to their highly customizable chemistry yielding programmable and unprecedented structures and properties. In particular, over the past decade, the synergistic relationship between the conductivity and porosity of 2D c-MOFs has paved the way toward their widespread applications. Despite their promising potential, the majority of 2D c-MOFs have yet to achieve atomically precise crystal structures, hindering the full understanding and control over their electronic structure and intrinsic charge transport characteristics. When modulating the charge transport properties of two-dimensional layered framework materials, decoupling the charge transport processes within and in between layers is of paramount importance, yet it represents a significant challenge. Unfortunately, 2D c-MOFs systems developed so far have failed to address such a major research target, which can be achieved solely by manipulating charge transport properties in 2D c-MOFs. 2D c-MOFs offer a significant advantage over organic radical molecules and covalent organic frameworks: polymerization through oxidative coordination is a viable route to form “spin-concentrated assemblies”. However, the role of these spin centers in charge transport processes is still poorly understood, and the intrinsic dynamics and properties of these spins have seldom been investigated. Consequently, overcoming these challenges is essential to unlock the full potential of 2D c-MOFs in electronics and other related fields, as a new type of quantum materials.

In this Account, we summarize and discuss our group’s efforts to achieve full control at the atomic level over the structure of 2D c-MOFs and their applications in electronics and spintronics, thereby providing distinct evidence on 2D c-MOFs as a promising platform for exploring novel quantum phenomena. First, we unravel the key role played by the rational design of the ligands to decrease the boundary defects, achieve atomically precise large single crystals, and investigate the intrinsic charge transport properties of 2D c-MOFs. The advantages and disadvantages of the current structural elucidation strategies will be discussed. Second, the fundamental challenge in 2D c-MOF charge transport studies is to decouple the in-plane and interlayer charge transport pathways and achieve precise tuning of the charge transport properties in 2D c-MOFs. To address this challenge, we propose a design concept for the second-generation conjugated ligands, termed “programmable conjugated ligands”, to replace the current first-generation ligands which lack modifiability as they mainly consist of *sp*^2^ hybridization atoms. Our efforts also extend to controlling the spin dynamics properties of 2D c-MOFs as “spin concentrated assemblies” using a bottom-up strategy.

We hope this Account provides enlightening fundamental insights and practical strategies to overcome the major challenges of 2D c-MOFs for electronics and spintronics. Through the rational design of structural modulation within the 2D plane and interlayer interactions, we are committed to making significant steps forward for boosting the functional complexity of this blooming family of materials, thereby opening clear perspectives toward their practical application in electronics with the ultimate goal of inspiring further development of 2D c-MOFs and unleashing their full potential as an emerging quantum material.

## Key References

- Lu, Y.; Zhong, H.; Li, J.; Dominic, A. M.; Hu, Y.; Gao, Z.; Jiao, Y.; Wu, M.; Qi, H.; Huang, C.; Wayment, L. J.; Kaiser, U.; Spiecker, E.; Weidinger, I. M.; Zhang, W.; Feng, X.; Dong, R. sp-Carbon Incorporated Conductive Metal–Organic Framework as Photocathode for Photoelectrochemical Hydrogen Generation. *Angew. Chem. Int. Ed.***2022**, *61* (39), e202208163.^1^*In this work, we synthesized the first sp-hybridized carbon embedded 2D c-MOF material and achieved atomically precise structural elucidation*.
- Lu, Y.; Zhang, Y.; Yang, C. Y.; Revuelta, S.; Qi, H.; Huang, C.; Jin, W.; Li, Z.; Vega-Mayoral, V.; Liu, Y.; Huang, X.; Pohl, D.; Polozij, M.; Zhou, S.; Canovas, E.; Heine, T.; Fabiano, S.; Feng, X.; Dong, R. Precise tuning of interlayer electronic coupling in layered conductive metal–organic frameworks. *Nat. Commun.***2022**, *13* (1), 7240.^2^*We proposed the design concept of the second generation conjugated ligands and used alkyl chain modification strategies to achieve precise regulation of the charge transport properties of 2D c-MOFs*.
- Lu, Y.; Hu, Z.; Petkov, P.; Fu, S.; Qi, H.; Huang, C.; Liu, Y.; Huang, X.; Wang, M.; Zhang, P.; Kaiser, U.; Bonn, M.; Wang, H. I.; Samori, P.; Coronado, E.; Dong, R.; Feng, X. Tunable Charge Transport and Spin Dynamics in Two-Dimensional Conjugated Metal–Organic Frameworks. *J. Am. Chem. Soc.***2024**, *146* (4), 2574–2582.^3^*This work reports the tuning spin dynamics through bottom-up rational design in 2D c-MOFs, and for the first time revealed that the main carriers of this type of material exist in spinless states*.

## Introduction

Two-dimensional conjugated metal–organic frameworks (2D c-MOFs) are an emerging class of electrically conductive organic 2D crystals with efficient in-plane conjugation and strong interlayer coupling that has recently garnered increasing attention and shown promise for various applications.^4−9^ 2D c-MOFs share structural similarities with graphite, consisting of layered structures with in-plane π-extended conjugation and out-of-plane π-orbital overlap, facilitating efficient charge transport within the 2D plane and along the π-stacking direction, as well as mass transport through ordered and aligned pores.^10,11^ These unique characteristics endow 2D c-MOFs with significantly enhanced electrical conductivities compared to conventional MOFs. Consequently, over the past decade, significant applications have arisen from the conductivity and porosity of 2D c-MOFs, including catalysis,^12^ energy storage,^13^ chemiresistive sensors,^14,15^ optoelectronic devices,^16^ and more.

**Figure 1:**
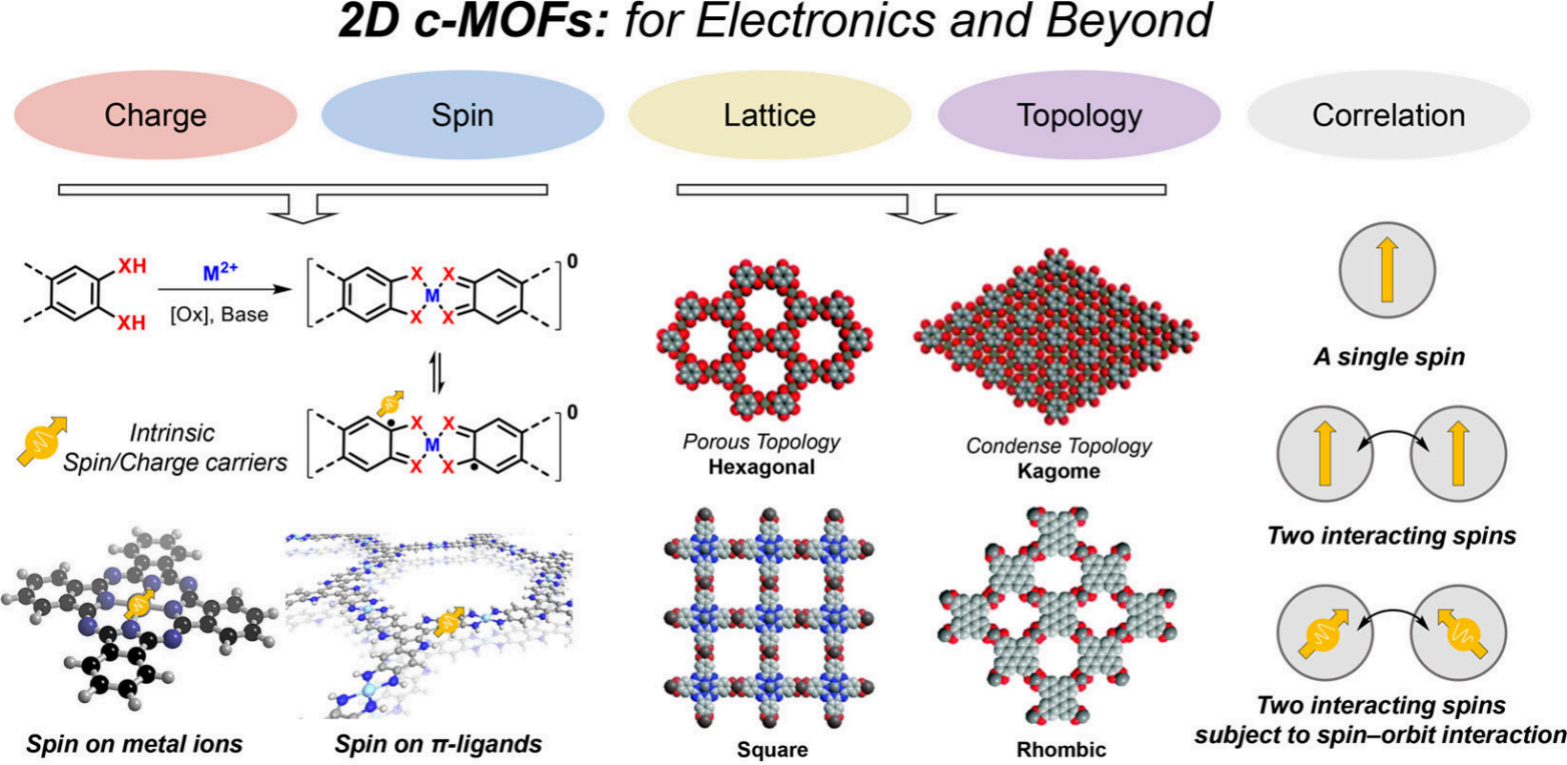
Scheme of 2D c-MOFs can serve as an ideal platform to study novel electronic, spin, and quantum properties.

Upon reevaluation, the fundamental uniqueness of 2D c-MOFs lies in their planar MX_4_ d-π conjugated Secondary Building Units (SBUs). The synthesis of 2D c-MOFs involves employing redox-driven coordination polymerization reactions to link organic conjugated ligands containing adjacent dihydroxy, diamino, or dithiol functionalities with metal ions, forming 2D d-π conjugated planes.^17,18^ These planes are subsequently assembled into three-dimensional bulk materials through interlayer π–π interactions. As a result, 2D c-MOFs possess intrinsic unpaired electrons located on the organic conjugated parts, realized by the redox coordination chemistry without additional doping. Alternatively, 2D c-MOFs can be viewed as the ordered assembly of organic conjugated molecules with molecular spins.^3,19^ Therefore, another implication of their conductive properties and porosity can be seen as molecular spins and topology, giving these materials their potential as new quantum materials. Molecular spin properties in 2D c-MOFs are influenced by in-plane chemical structures and interlayer interactions, while their topological structures are governed by the symmetry and topology of the π-conjugated aromatic monomers used in their construction. 2D c-MOFs could be regarded as spin-concentrated molecular assemblies, offering the opportunity to systematically optimize spin–lattice and spin–spin interactions compared to isolated organic conjugated molecules. Consequently, the intrinsic *charge* and *spin* of 2D c-MOFs can enable unique *correlation* through the *lattice* and *topology*, which in principle can serve as an ideal platform to study novel quantum properties (Figure 1), such as topological insulators, superconducting, quantum computing, and spin liquids, particularly in the emerging fields of spintronics and quantum information science.^3,19−22^ However, current studies primarily focus on the charge transport properties, while less attention has been devoted to exploring the properties of 2D c-MOFs beyond electronics.

In this Account, we will outline our strategies to tackle the key challenges faced currently by the 2D c-MOFs research community, focusing mainly on their properties and applications beyond electronics. First, we will discuss the importance of single crystal structures of 2D c-MOFs for understanding their intrinsic electronic properties. Next, we will explore the utilization of alkyl chain substitutions to achieve precise editing of conjugated ligands at the molecular level, thereby effectively regulating their charge transport properties between layers. Finally, we will delve into controlling the spin dynamics properties of 2D c-MOFs, paving the way for their applications in spintronics and quantum materials.

## Challenge 1: Making Large Single Crystals of 2D c-MOFs

At present, the majority of 2D c-MOFs encounter challenges in obtaining an atomically precise structural analysis, severely limiting their understanding in electronics, spintronics, and quantum properties.^23,24^ Minor changes in the chemical structure of 2D c-MOFs can have significant effects on their electronic structure, impeding our ability to establish reliable structure–property relationships.^25^

By operating under thermodynamic control, the primary approach to overcoming this challenge is to master and exploit the reversible nature of coordination polymerization reactions during 2D c-MOF synthesis to enhance crystallinity and crystal size, combining this with various sophisticated structural characterization techniques, such as diffraction methods and electron microscopy imaging-based approaches, to gain insight into the material’s structure as accurately as possible. Significant control over the rate of coordination polymerization and enhancement of reversibility can be achieved by tuning the reactivity and reversibility of coordination reactions, leading to enhanced crystallinity of the materials.

**Figure 2:**
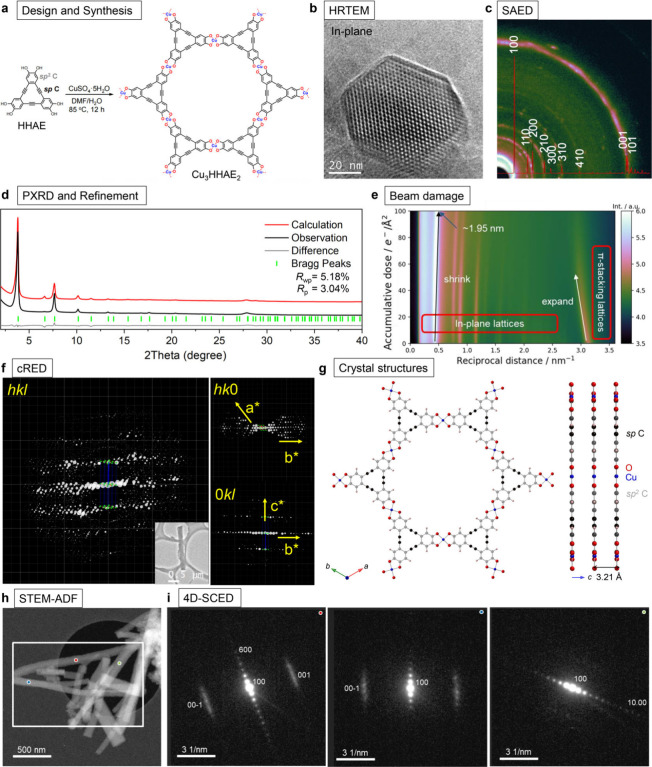
(a) Synthetic scheme of Cu_3_HHAE_2_. (b) HRTEM image of a Cu_3_HHAE_2_. (c) Zero-loss filtered selection area electron diffraction (SAED) pattern of Cu_3_HHAE_2_ with simulated powder pattern superimposed. (d) Overlay of the experimental and Rietveld refinement plots of Cu_3_HHAE_2_. (e) Evaluation of 300 kV electron beam damage of Cu_3_HHAE_2_ at room temperature. Azimuth integrated zero-loss filtered SAED (horizontal axis) as a function of accumulative electron dose. (f) Projection of 3D reciprocal lattice reconstructed cRED data. (g) Portion of the crystal structures. (h) Scanning transmission electron microscope annular dark-field (STEM-ADF) image of the sample (acquired after diffraction imaging). White box region indicates (i) 4D-scanning confocal electron diffraction (4D-SCED) data were acquired. Adapted with permission from ref (1). Copyright 2022 Wiley-VCH Verlag GmbH & Co.

Here, we present the structural elucidation of the first *sp* carbon-embedded 2D c-MOF, Cu_3_HHAE_2_, through a coordination reaction between a hexahydroxyarylene-ethynyl macrocycle ligand (HHAE)—the smallest graphdiyne unit—and Cu^2+^ salt (Figure 2a). The introduction of electron-deficient *sp*-carbons can decrease the electron density at the metal-binding site, thereby increasing the acidity of the coordination group (−OH) and enhancing the reversibility of the metal–ligand bond during MOF growth. Scanning electron microscopy (SEM) revealed that Cu_3_HHAE_2_ exhibits hexagonal rods at the scale of 1–5 μm, indicating the achievement of high-quality single crystals.

Initially, continuous rotation electron diffraction (cRED) technique was employed on Cu_3_HHAE_2_ crystals to determine its structure.^26^ From the 3D reciprocal lattice constructed by the RED data set (Figure 2f), a set of estimated unit cells was obtained. The latter reveals that Cu_3_HHAE_2_ adopts a honeycomb-like arrangement through the coordination between Cu ions and HHAE units in a 3:2 ratio. Notably, Cu_3_HHAE_2_ crystallizes in a perfect hexagonal structure, belonging to the *P*6/*mmm* space group, with unit cell dimensions of a = b = 26.54 Å, c = 3.21 Å, and angles α = β = 90.0°, γ = 120.0° (Figure 2g). Moreover, powder X-ray diffraction (PXRD) analysis of Cu_3_HHAE_2_ reveals distinct peaks at 2θ = 3.8°, 7.6°, 10.1°, 13.3°, 17.6°, and 20.3°, indicating long-range order within the ab plane (Figure 2d). Additionally, a peak at *2θ* = 27.9° corresponds to the π–π stacking diffraction. The powder refinement of Cu_3_HHAE_2_ yields *R*_p_ = 3.04% and *R*_wp_ = 5.18%, demonstrating the convergence of experimental data. Importantly, the experimental PXRD data aligns perfectly with the calculated XRD pattern, confirming the high phase purity of the Cu_3_HHAE_2_ sample. This phase purity analysis is crucial for structural elucidation, as the structure revealed by cRED represents only that of a small individual crystal.

High-resolution transmission electron microscopy (HRTEM) lattice images captured from small crystallites oriented near the {001} zone axis (Figure 2b) directly reveal a honeycomb-like arrangement with a pore size of 2.1 nm, consistent with the single-crystal structures. Additionally, experimental elastically filtered selection area electron diffraction (SAED) shows excellent agreement with simulations using the structural model derived previously (Figure 2c). To confirm the orientation of the nanorods, representative patterns extracted from the color-dotted positions are shown as insets in Figure 2h-i, demonstrating a remarkably high degree of crystallinity: up to {12.00} diffraction spots are visible in the raw data. Across all probed regions, the diffraction vector {001} coincides with the long axis of the rods, while the {100} diffraction arranges along the short axis, consistent with HRTEM observations and confirming that the pores align along the long axis of the rods. Furthermore, the beam damage process of Cu_3_HHAE_2_ at room temperature was characterized (Figure 2e). As the electron beam dose accumulates, the in-plane lattice connected by coordination bonds contracts, while the out-of-plane stacking direction mediated by π–π interactions expands. These findings are pivotal for characterizing 2D c-MOFs using electron beam-related techniques and directly influence the accuracy and resolution of characterization. The structural characterization by combining XRD, electron diffraction, and microscopy provides unambiguous evidence that these 2D sheets form bulk hexagonal rods in perfect eclipsed stacking. At this point, we can confidently affirm that we have obtained the reliable crystal structure of Cu_3_HHAE_2_, laying the foundation for understanding its intrinsic electronic properties.

**Figure 3:**
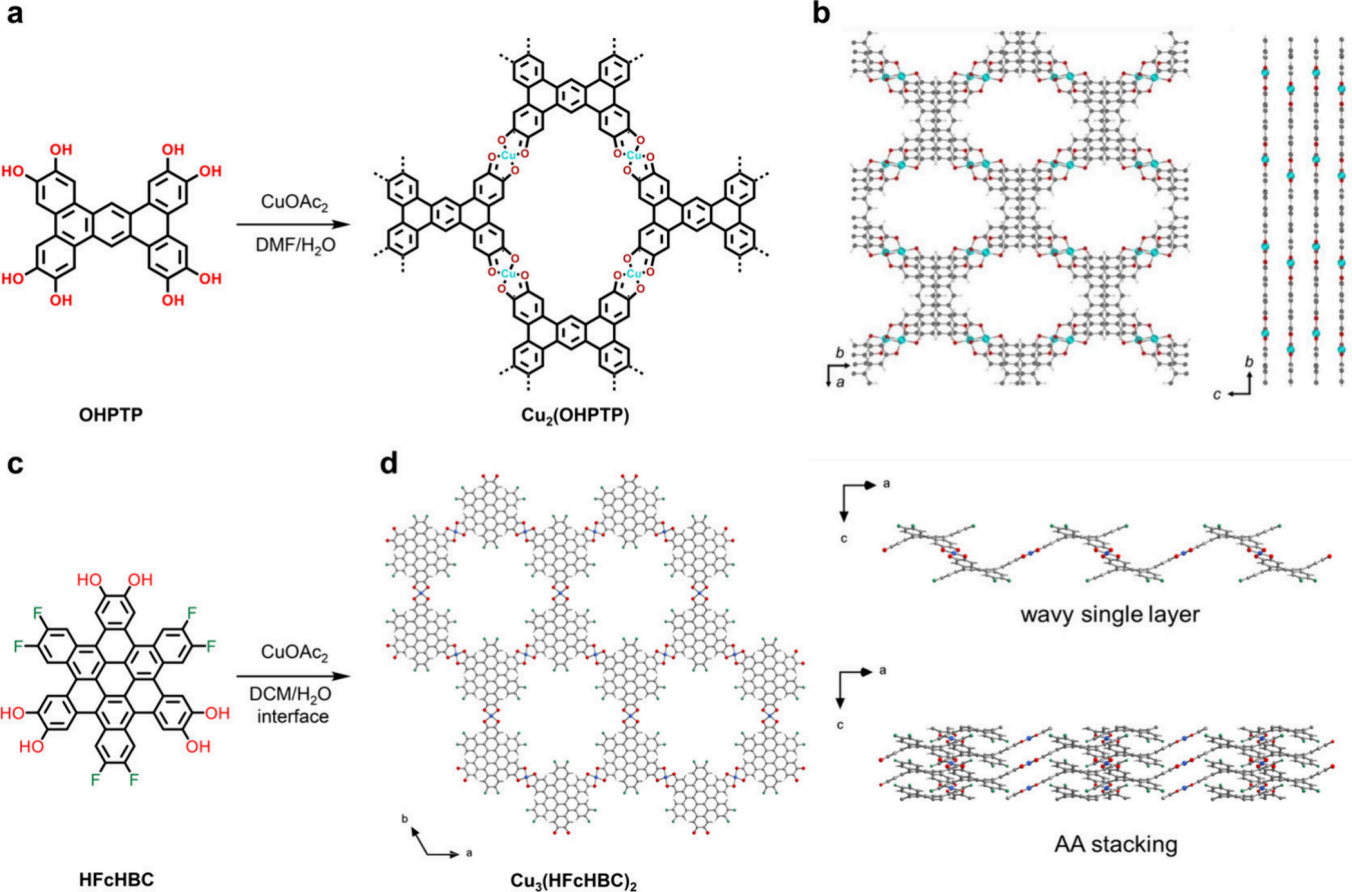
(a) Synthetic Scheme of Cu_2_(OHPTP). (b) In-plane and out-of-plane of Cu_2_(OHPTP) structure models, respectively. Adapted with permission from ref (37). Copyright 2023 Wiley-VCH Verlag GmbH & Co. (c and d) Synthetic Scheme and the portion of the Cu_3_(HFcHBC)_2_ crystal structure. C, O, Cu, F, and H atoms are shown in gray, red, blue, green, and white, respectively. Reprinted with permission from ref (28). Copyright 2023 American Chemical Society.

Enhancing the π–π stacking interaction while preserving the reversibility of the 2D in-plane coordination reaction stands out as a crucial strategy to improve the crystal quality of 2D c-MOFs. We embarked on designing a novel π-extended ligand, phenanthrotriphenylene (OHPTP) with *D*_2h_ symmetry (Figure 3a).^27^ Through our synthetic efforts, we successfully produced the first rhombic 2D c-MOF single crystals, denoted as Cu_2_(OHPTP) (Figure 3b). Detailed analysis using cRED unveiled the orthorhombic crystal structure at the atomic level, featuring a distinctive slipped AA stacking arrangement. This arrangement involves a shift of two layers along the *b*-axis, with an offset nearing 2 Å. Such a slipped AA stacking configuration fosters an overlap of the aromatic cores while effectively preventing the direct stacking of Cu atoms. Consequently, the positioning of the metal nodes induces a distorted pseudo-octahedral coordination environment for the Cu atoms. Theoretical calculations underscore the significant contribution of out-of-plane charge transport in this semiquinone-based 2D c-MOF, emphasizing the intricate interplay between molecular design and structural topology in dictating electronic properties.

Hitherto, all conjugated ligands that have been reported are based on planar conjugated polycyclic aromatic hydrocarbons (PAHs). Compared with the planar PAHs, the nonplanar PAHs can change the orbital structures and electron densities between concave and convex faces, which causes concave and convex face act as acceptors and donors, respectively. Therefore, curved molecular surfaces prefer to interact with curvature-similar molecules, which is called self-complementarity. Nonplanar/curved π-conjugated PAHs are advantageous to guide the arrangement of layers into eclipsed stacking due to their Janus character, which results in the formation of highly crystalline wavy-shaped 2D c-MOF, thus rendering a long-range charge transport along the stacking direction. To address this point, we introduced the synthesis and characterization of a novel 2D c-MOF, designated as Cu_3_(HFcHBC)_2_, featuring a fluorinated core-twisted contorted hexahydroxy-hexa-*cata*-hexabenzocoronene (HFcHBC) ligand (Figure 3c-d).^28^ The crystal structure is elucidated through HRTEM and cRED, revealing a distinctive wavy honeycomb lattice arrangement with AA-eclipsed stacking. Theoretical calculations suggest that Cu_3_(HFcHBC)_2_ possesses a metallic state. However, experimental analysis of crystalline film samples, which contain numerous grain boundaries, manifests semiconducting behavior on a macroscopic scale, characterized by discernible thermally activated conductivity. Temperature-dependent electrical conductivity measurements conducted on isolated single-crystal devices corroborate the metallic nature of Cu_3_(HFcHBC)_2_, albeit exhibiting minor thermally activated transport behavior.

Although current high-throughput computing and advanced theoretical simulations can accurately predict the structures of many framework materials, predicting the structures of 2D c-MOFs assembled by noncovalent interactions, such as π–π interactions, remains quite challenging due to the complexity of these interactions.^29^ Specifically, while the chemical structure of conjugated ligands and SBUs can accurately predict the monolayer structure of 2D c-MOFs, accurately predicting the stacking patterns of these monolayers when forming bulk materials is difficult. These differences can be identified by comparing the precise crystal structures of the same 2D c-MOF with the results of theoretical simulations.^30−32^ In 2D c-MOFs interconnected by well-defined SBUs (e.g., CuO_4_), these distinctions primarily stem from stacking faults between layers. Identifying the source of these differences becomes more complex when it comes to SBUs that are not precisely resolved. For example, the crystal structure of 2D c-MOFs formed by Zn ions and catechol ligands (e.g., ZnHHTP) has yet to be elucidated at atomic levels, and the high stability of ZnHHTP under redox conditions suggests that its SBUs may not conform to classic planar tetragonal structures. However, even slight deviations between the simulated and actual structures can undeniably trigger a “butterfly effect” on the material’s band structures—affecting band dispersion, Fermi levels, and effective carrier masses—thereby impeding the establishment of reliable structure–property relationships.

## Challenge 2: Decoupling the In-Plane and Interlayer Charge Transport Properties in 2D c-MOFs

**Figure 4:**
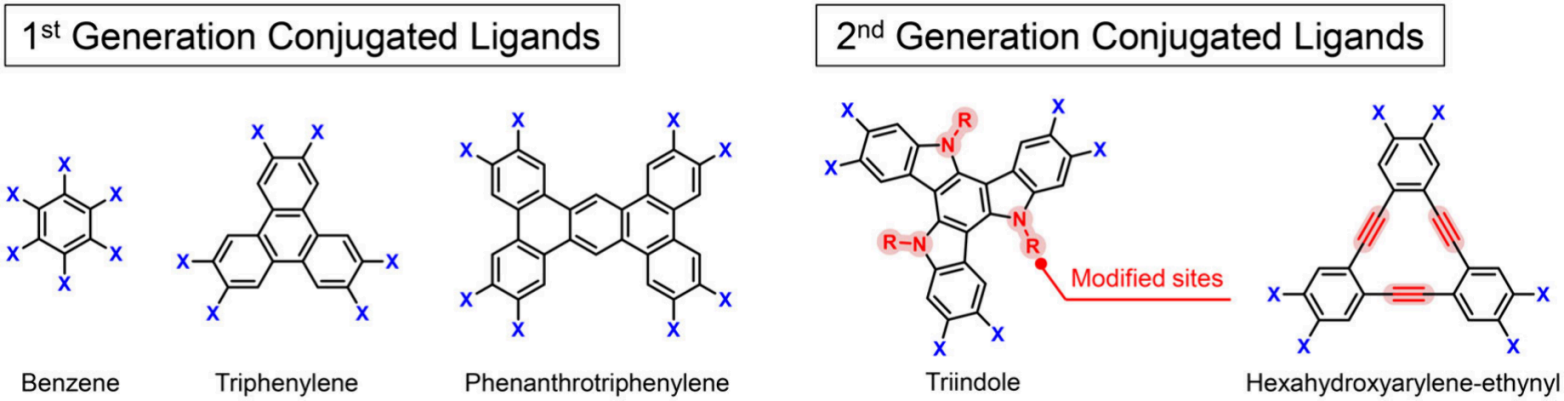
Representative 1st and 2nd generation conjugated ligands for 2D c-MOFs. X represents the −OH, −NH_2_, −SH et al. coordination groups, and R represents the functional groups.

Conductivity is the most important characteristic that distinguishes 2D c-MOFs from other framework materials. Therefore, understanding their charge transport mechanism and precisely controlling their charge transport properties are crucial for the practical application in electronic devices. During the past decade, significant efforts have been made to modify the charge transport properties by tuning the intralayer π-extended conjugation of 2D c-MOFs. This has been achieved by designing the topological networks and varying the chemical structures of the ligands (e.g., varying the size and symmetry of π-conjugated building block) (Figure 4),^23,33−35^ as well as by tailoring the composition of the SBUs (e.g., metal ions and coordination functional groups).^36^ However, for 2D c-MOFs with efficient charge transport pathways through both in-plane and out-of-plane directions, these modulation strategies cannot decouple their respective contributions. The fundamental reason for this is that when adjusting one of the aforementioned variables, there is a “coupled” change in both in-plane and out-of-plane electronic structure.

**Figure 5:**
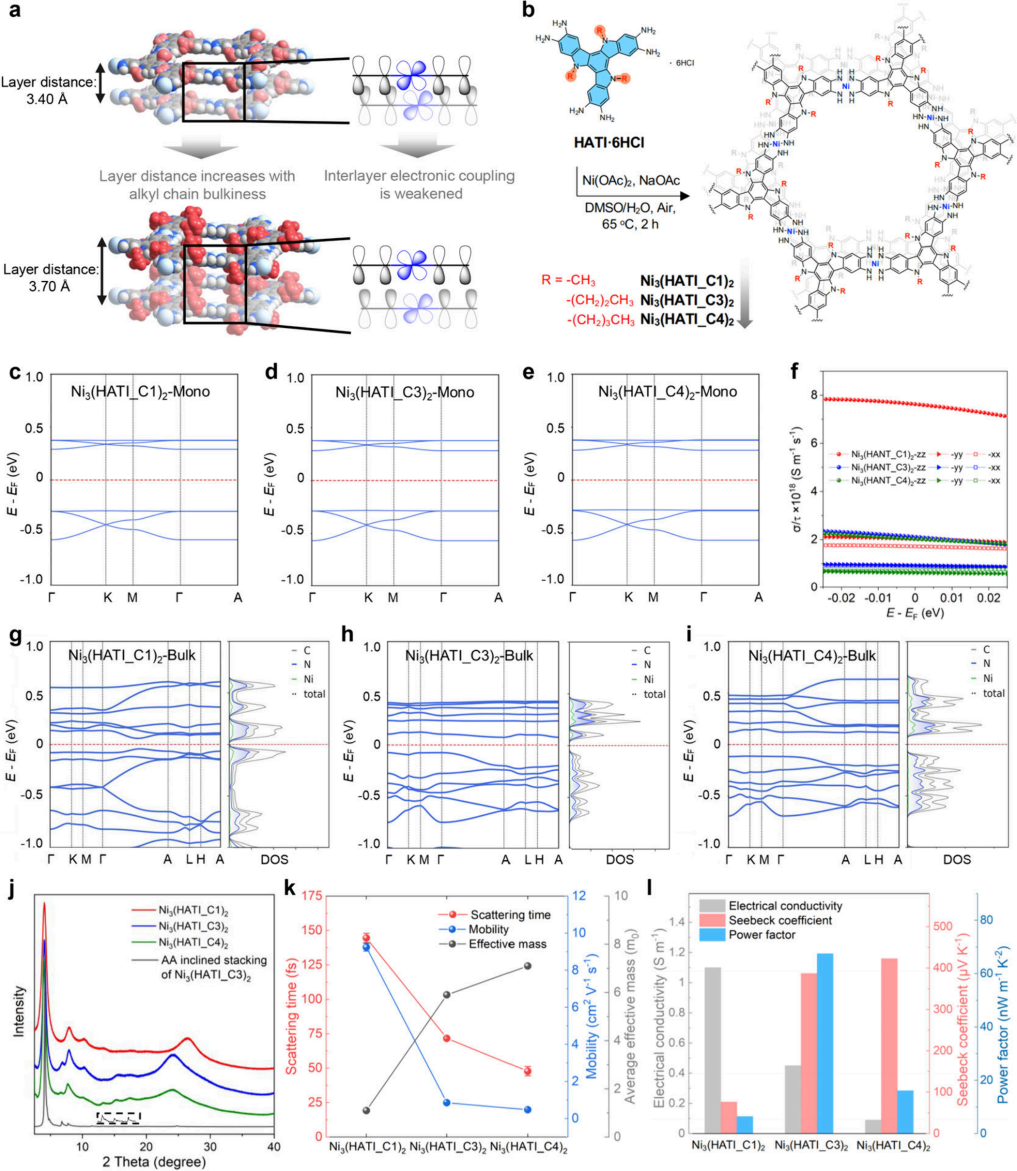
(a) Schematic diagram of the concept of the precise control of the interlayer electronic coupling. red balls: alkyl chains; gray balls: C; white balls: H; blue balls: N; cyan balls: Ni. (b) Synthesis of Ni_3_(HATI_CX)_2_ with different lengths of alkyl chains. (c–e) The calculated band structures of Ni_3_(HATI_CX)_2_ monolayer. (f) Calculated electrical conductivity within the constant-relaxation-time approximation of the Boltzmann transport equation. (g–i) The calculated band structures and projected density of states (PDOS) of bulk Ni_3_(HATI_C1)_2_. (j) PXRD patterns for Ni_3_(HATI_CX)_2_ and calculated PXRD patterns of Ni_3_(HATI_C3)_2_ with AA-inclined stacking modes. (k) Scattering time, mobility, and average effective mass of Ni_3_(HATI_CX)_2_. (l) The thermoelectric properties (electrical conductivity, Seebeck coefficient, and power factor) of Ni_3_(HATI_CX)_2_ pellets. Reproduced with permission from ref (2). Copyright The Authors, some rights reserved; exclusive licensee Springer Nature. Distributed under a Creative Commons Attribution License 4.0 (CC BY).

To overcome this challenge, we have designed a novel class of conjugated ligands, 2,3,7,8,12,13-hexaiminotriindole (HATI) ligand, which enables the modification of different functional groups at the nitrogen atoms of indole.^2^ The highly tunable nature of HATI distinguishes itself from traditional conjugated ligands represented by benzene, triphenylene, and phthalocyanine, paving the way toward the second-generation conjugated ligands that can be precisely edited at the molecular level (Figure 4). To achieve the decoupling of in-plane and interlayer charge transport, we initially modified HATI by attaching the alkyl chains with varying steric bulkiness (methyl, n-propyl, and *n*-butyl groups, denoted as C1, C3, and C4, respectively) (Figure 5a,b). As alkyl chains possess insulating properties, they only affect the van der Waals interactions between layers, leaving the electronic structure of the 2D conjugated plane unaltered.

Subsequently, we synthesized a series of triindole-based 2D c-MOFs with identical SBUs using a solvothermal method. The major PXRD diffraction peaks observed in the low-angle region are consistent across the three resulting 2D c-MOFs, indicating consistent in-plane crystalline ordering despite variations in alkyl chain lengths. The PXRD patterns reveal interlayer spacing values of 3.40, 3.68, and 3.70 Å for Ni_3_(HATI_C1)_2_, Ni_3_(HATI_C3)_2_, and Ni_3_(HATI_C4)_2_, respectively (Figure 5j). Within these 2D c-MOFs, the interlayer distance gradually increases upon lengthening the alkyl chains from C1 to C4. It should be noted that despite our extensive synthetic efforts, we have not yet obtained sufficiently high-quality crystals suitable to perform an accurate structural analysis with the cRED method. This difficulty underscores one of the significant obstacles faced by amino-based conjugated ligands, arising from the rapid reactions between transition metal ions and o-phenylenediamine-derived ligands.^37^

Density functional theory (DFT) calculations suggest that the monolayers of Ni_3_(HATI_CX)_2_ with varying lengths of alkyl chains exhibit similar band dispersions and band gaps (Figure 5c-e). Thus, the addition of alkyl chains of different lengths has minimal influence on the charge transport properties within the 2D plane of Ni_3_(HATI_CX)_2_. In the band structures of layer-stacked Ni_3_(HATI_CX)_2_, the inclusion of various alkyl chains indirectly affects out-of-plane electronic coupling by adjusting the interlayer spacing. The increased effective masses from Ni_3_(HATI_C1)_2_ to Ni_3_(HATI_C4)_2_ indicate a decrease in electronic coupling upon increasing the alkyl chain length (Figure 5g–i).

The room-temperature electrical conductivities of the powder pellets were measured as ∼1.1 S m^–1^, 0.45 S m^–1^, and 0.09 S m^–1^ for Ni_3_(HATI_C1)_2_, Ni_3_(HATI_C3)_2_, and Ni_3_(HATI_C4)_2_, respectively. The electrical conductivities of Ni_3_(HATI_CX)_2_ were found to decrease by over 1 order of magnitude upon increasing the interlayer distance. Subsequently, terahertz time-domain spectroscopy (THz-TDS) measurements were conducted at room temperature in a dark environment to elucidate the charge transport properties of these 2D c-MOFs. Consequently, these 2D c-MOFs demonstrate carrier mobility values of 9.2 ± 0.2, 0.9 ± 0.01, and 0.4 ± 0.03 cm^2^ V^–1^ s^–1^ for Ni_3_(HATI_C1)_2_, Ni_3_(HATI_C3)_2_, and Ni_3_(HATI_C4)_2_, respectively (Figure 5k). The plot of conductivity at constant relaxation time shows that the difference between interlayer conductivity and intralayer conductivity gradually decreases with increasing interlayer distance, with in-plane conductivity being consistently lower than interlayer conductivity for all three cases (Figure 5f). Our findings indicate that a shorter interlayer spacing between consecutive 2D c-MOF planes enhances the charge carrier mobility and, consequently, the electrical conductivity. We refer to this phenomenon as “layer-dependent” charge transport properties in 2D c-MOFs.

Even more captivating is the utilization of strategies to control the interlayer electronic coupling, which can balance the conductivity and Seebeck coefficient of 2D c-MOFs, thus tuning their thermoelectric performance.^38^ The measured Seebeck coefficients are 76.3 ± 2, 387.2 ± 2, and 423.8 ± 1 μV K^–1^ for Ni_3_(HATI_C1)_2_, Ni_3_(HATI_C3)_2_, and Ni_3_(HATI_C4)_2_, respectively. The considerable disparity in Seebeck coefficients among the Ni_3_(HATI_CX)_2_ samples likely stems from differences in band structures and effective mass values. Remarkably, the combination of electrical conductivity and Seebeck coefficient yields a thermoelectric power factor (*PF*) as high as 68 ± 3 nW m^–1^ K^–2^ for Ni_3_(HATI_C3)_2_ at room temperature, providing a record high power factor value among reported p-type MOFs (Figure 5l).

In the rational design of 2D c-MOFs, the scarcity of planar MX_4_ SBUs underscores the crucial importance of original innovation in organic conjugated ligands. The core concept behind the design of the second-generation organic conjugated ligands, exemplified by HATI, lies in introducing heteroatoms or other non-*sp*^2^ hybridized carbon atoms into the conjugated framework.^1,32,39^ This strategy allows for precise molecular editing of the conjugated ligand either before or after the synthesis of 2D c-MOFs, paving the way for precise control over the electronic and other relevant properties. For instance, incorporating diverse functional groups onto conjugated ligands prior to coordinating polymerization can yield a range of topologically analogous conjugated ligands. This strategy enables the dissociation of charge transport in the plane and perpendicular to it while preserving the same in-plane connectivity. Moreover, postformation of 2D c-MOFs, pre-embedded stimuli-responsive functional groups can be exploited to elicit responses to diverse ions (including metal ions and protons), thereby enabling regulation of carrier concentration and mobility.^32,39^ This innovative design principle for conjugated ligands not only facilitates accurate adjustment of charge transport characteristics but also lays the groundwork for practical electronic device applications of 2D c-MOFs. It offers potential solutions to enhance solution processability by increasing the solubility of the conjugated frameworks.

## Challenge 3: Manipulating the Spin Dynamics in 2D c-MOFs

Despite its foundational significance in spintronics and quantum materials, achieving systematic control over the molecular spin dynamics in 2D c-MOFs through bottom-up molecular design has yet to be realized. The dynamics of molecular spins are primarily dictated by the chemical environment surrounding the spin.^40−43^ In the case of 2D c-MOFs, molecular spin has the possibility to delocalize throughout the entire ligand but is primarily localized on the carbon atoms bonded to the coordinating atoms. Thus, for a single layer of 2D c-MOFs, the first local connection sphere of this spin is determined by the coordinating atoms and the atoms within the adjacent conjugated framework, while the second connection sphere is influenced by the metal ions and the substituents on the adjacent carbon atoms.^19,44^ As the single layer assembles into bulk materials, the interactions between layers also impact the chemical environment surrounding the molecular spin. Nevertheless, the majority of 2D c-MOFs demonstrate a nearly fully overlapping stacking, resulting in typical interlayer distances of 3.2–3.3 Å due to out-of-plane π–π interactions;^23,24,30,45^ such strong interlayer interactions lead to a decrease in the density of active spins and accelerate spin–lattice relaxation and spin decoherence in 2D c-MOFs.

**Figure 6:**
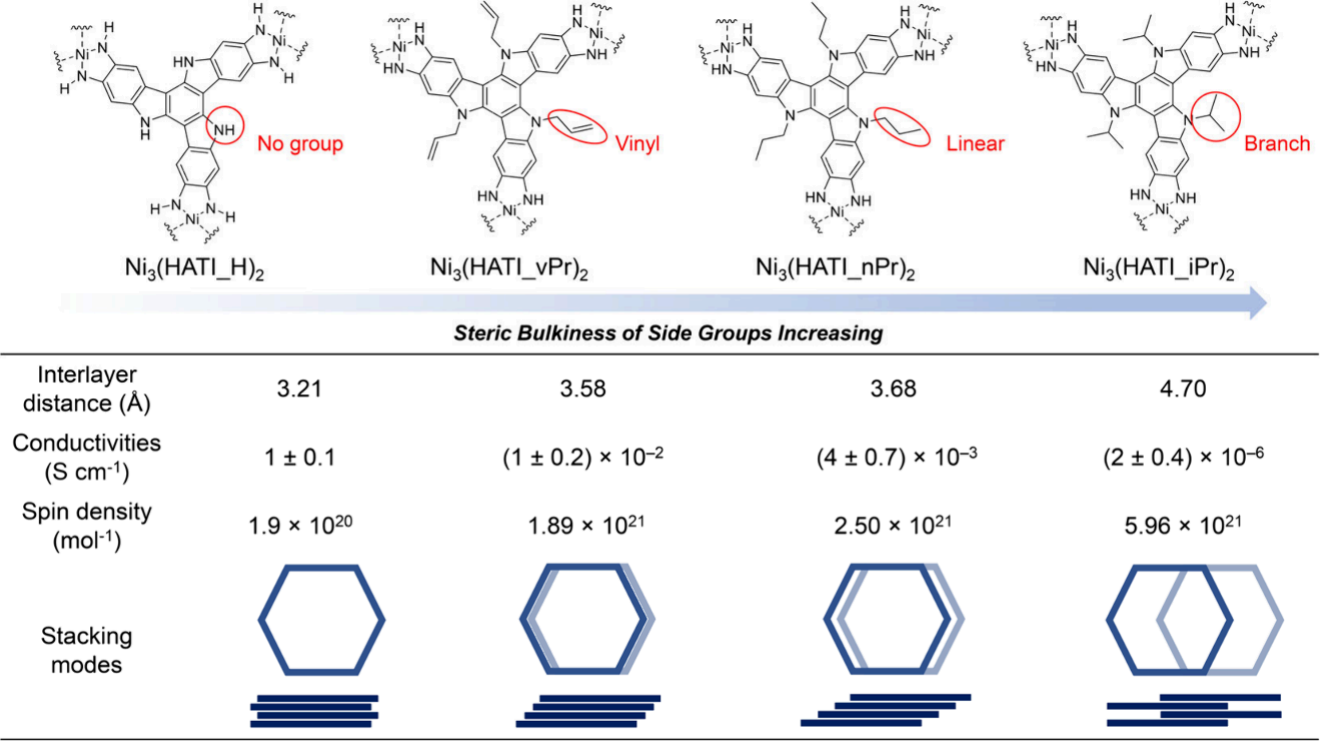
Summary of the results about the structural information, electrical conductivities and spin densities at room temperature of Ni_3_(HATI_X)_2_.

As layered van der Waals materials, spin communication within 2D c-MOFs heavily relies on the spatial arrangement, such as stacking modes, of the 2D layers. The stacking modes can be controlled by leveraging noncovalent interactions between the layers. Hence, we envision that introducing side groups of variable sizes onto the conjugated ligands might serve as the structural perturbation to modulate interlayer interactions, thereby tuning spin dynamics in 2D c-MOFs (Figure 6). Herein, we introduced different types of side groups, including hydrogen atom, allyl, n-propyl, and isopropyl (referred to as H, vPr, nPr, iPr, respectively), grafted on HATI ligands. After integrating PXRD, HRTEM, and pore size distribution analyses to characterize the structure, we discovered that the varying steric bulkiness of these side groups enables the synthesized 2D c-MOFs (Ni_3_(HATI_X)_2_) to exhibit significant variations in interlayer arrangement, despite sharing identical SBUs. Ni_3_(HATI_H)_2_ showed an AA-serrated stacking model with an interlayer distance of 3.20 Å. Ni_3_(HATI_vPr)_2_ and Ni_3_(HATI_nPr)_2_ exhibited the isomorphic structures of AA-inclined stacking, and due to the smaller steric bulkiness of the unsaturated allyl group, Ni_3_(HATI_vPr)_2_ possesses a closer interlayer distance (3.58 Å) when compared to saturated propyl substituted Ni_3_(HATI_nPr)_2_ (3.68 Å). Conversely, the isopropyl-substituted Ni_3_(HATI_iPr)_2_ adopts a distinct staggered stacking to alleviate repulsion between the side groups, yielding the largest interlayer distance of around 4.70 Å among this family of 2D c-MOFs, which also indicates a significantly reduced interlayer interaction. Notably, the electrical conductivity of these 2D c-MOFs gradually decreases from 1 to 10^–6^ S cm^–1^ with side group bulkiness increasing.

**Figure 7:**
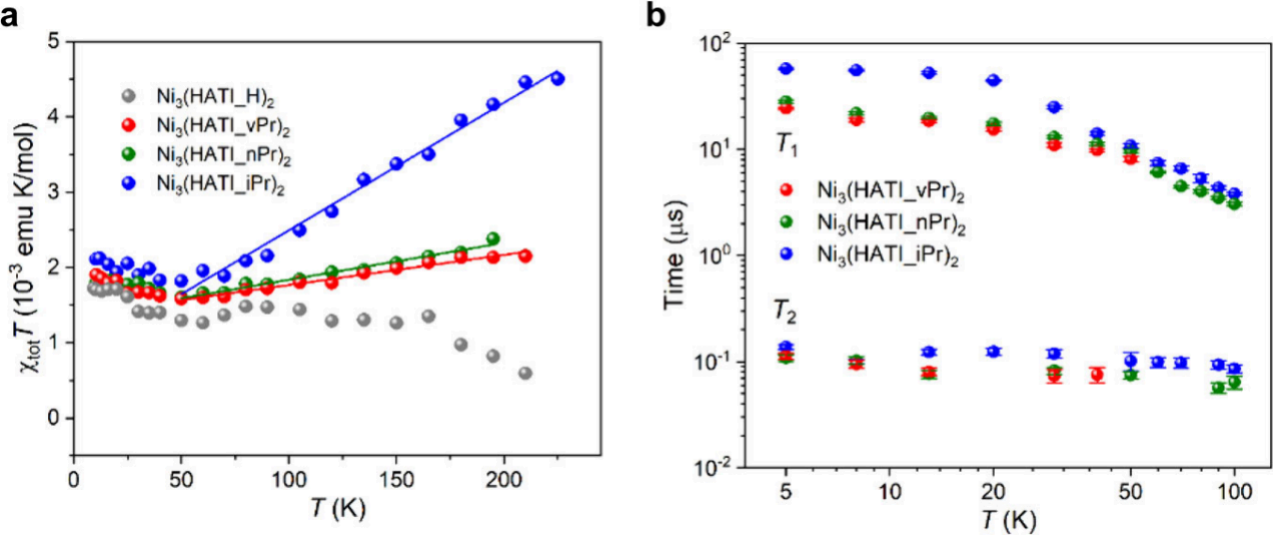
(a) χ_tot_*T* versus temperature (*T*) plot for the spin susceptibility (χ_tot_) obtained from the double integration of the ESR spectra. The solid lines are fits using χ_tot_*T* = *C* + χ_pauli_*T*, where *C* and χ_pauli_ are the Curie constant and Pauli susceptibility. (b) Temperature dependence of *T*_1_ and *T*_2_ for Ni_3_(HATI_vPr)_2_, Ni_3_(HATI_nPr)_2_, and Ni_3_(HATI_iPr)_2_. Reprinted with permission from ref (3). Copyright 2024 American Chemical Society.

To reveal which charge carriers play a key role in charge transport in these 2D c-MOFs, the temperature dependence of spin susceptibility (χ_tot_) is extracted from double integration of the electron spin resonance (ESR) spectra. The net susceptibility is written as a sum of Pauli and Curie susceptibilities, χ_tot_ = χ_Pauli_ + χ_Curie_, referring to free and localized electrons, respectively.^46^ These two contributions can be better presented in a χ_tot_*T* – *T* plot by χ_tot_*T* = χ_Pauli_*T* + *C*, where the *C* is Curie constant. For Ni_3_(HATI_vPr)_2_, Ni_3_(HATI_nPr)_2_, and Ni_3_(HATI_iPr)_2_, clear slopes are observed in the temperature region of 50–200 K, which might indicate Pauli contributions from free conduction electrons (Figure 7a). On the contrary, the most conductive Ni_3_(HATI_H)_2_, shows almost constant χ_tot_*T* values as temperature increases, and thus no noticeable line broadening is observed. The existence of free electrons makes the materials susceptible to the Elliott mechanism.^47,48^ Overall, these analyses allow us to conclude that higher conductivity might lead to less free electrons within these MOF materials. This rather counterintuitive behavior is nevertheless not unseen, and suggests that the carrier transport in these 2D c-MOFs is dominated by spinless polaron pairs or bipolarons, which seems to be contrary to the currently widely accepted theory that the organic radicals in 2D c-MOFs play the role of carriers.

Attaining a high spin density while maintaining a prolonged spin relaxation time poses a paramount challenge for spin-concentrated assemblies. In conventional spin assemblies, the dipole interaction among electron spins escalates notably with increasing electron density, thereby markedly hastening the spin relaxation process.^49^ For the Ni_3_(HATI_X)_2_ system, the spin densities should be identical among the samples, as the spins in these 2D c-MOFs originate from the SBUs formed by the oxidative coordination reaction between Ni ions and o-phenylenediamine derivatives. The modification of saturated alkyl chains is not expected to influence the spin densities of these 2D c-MOFs. Ni_3_(HATI_H)_2_, Ni_3_(HATI_vPr)_2_, Ni_3_(HATI_nPr)_2_, and Ni_3_(HATI_iPr)_2_ present spin densities of 1.9 × 10^20^, 1.89 × 10^21^, 2.50 × 10^21^, and 5.96 × 10^21^ mol^–1^, obtained by double integration of room-temperature ESR results, respectively (Figure 6). Substitution of bulky side groups (branching alkyl chains) on conjugated ligands will dislocate the 2D layer and expand the interlayer distance of 2D c-MOFs, which would spatially reduce the coupling of the spin between the two layers. As a result, the spin density of Ni_3_(HATI_iPr)_2_ is 30 times higher than that of Ni_3_(HATI_H)_2_.

Pulsed ESR measurements enable to further probe the spin–lattice relaxation time (*T*_1_) and spin decoherence time (*T*_2_) of Ni_3_(HATI_X)_2_ (Figure 7b). We found that Ni_3_(HATI_iPr)_2_ exhibits substantially longer *T*_1_ of up to ∼60 μs than Ni_3_(HATI_H)_2_, where spin relaxations occur too rapidly to be detected. This is likely due to the greater interlayer distance in Ni_3_(HATI_iPr)_2_ resulting from the most sterically demanding side group and the mismatched stacking mode, which might give rise to an ultraweak interlayer interaction and hinders the lattice phonons. The ultrashort spin–lattice relaxation time of Ni_3_(HATI_H)_2_ might be attributed to the scattering of the metal-like conduction electrons by a phonon. For the *T*_2_, the values (∼100 ns) are comparable among different samples, which can be explained by the chemical similarities in these materials with the similar π-conjugated structure and nuclear spin bath, and the fact that communication between spins may mainly occur within the 2D layer. Therefore, further improvements should be anticipated with the aim of excluding nuclear spin-rich atoms, such as nitrogen and hydrogen, while maintaining the weak interlayer interactions in 2D c-MOFs.

Based on these findings, we can affirm that 2D c-MOFs represent a novel material platform for exploring spintronics and quantum exotic states. The primary objective now is to establish a reliable structure–property relationship, which can be divided into three key levels. (1) At the molecular level, this entails the selection and design of SBUs, encompassing metal ions and coordinating functional groups. (2) It extends to the modulation of the 2D plane, including the chemical structure, symmetry, and modifying groups of conjugated ligands, which directly influence the topological structure of the 2D plane, thereby affecting spin-mediated interactions and correlations. (3) Achieving full control over the effect of the stacking modes between layers.

## Conclusion and Perspective

In summary, in view of their intrinsic charge carriers, persistent molecular spins, and highly tunable topological structures, 2D c-MOFs have established themselves as an exciting emerging class of materials holding an enormous potential for technological application in electronics, spintronics, and quantum information science. This report outlines three main obstacles that 2D c-MOFs face in evolving toward next-generation electronic and quantum materials, and we approach these challenges from the following aspects by rationally constructing 2D c-MOFs: (1) ligand structure and topology, (2) ligand functional groups, and (3) 2D plane stacking modes; these three aspects play the critical roles on the electronic structure, charge transport properties, and spin dynamics properties of 2D c-MOFs. Specifically, making high-quality crystals through rational conjugated ligands design of the 2D c-MOFs forms the basis for exploring their electronic and spin-related applications. We achieved precise structural analysis at the atomic level by developing the *sp* carbon-embedded conjugated ligands, extended π-conjugated ligands, and the nonplanar conjugated ligands. Furthermore, we introduced design principles enabling the emergence of the second-generation organic conjugated ligands, employing precise molecular editing strategies, such as the alkyl chain effect, to achieve precise control over the charge transport properties of 2D c-MOFs. Additionally, we demonstrated the broader range of control over interlayer interactions in 2D c-MOFs using alkyl chain strategies, enabling efficient manipulation of spin dynamics. Overall, our efforts have addressed existing limitations and provided a series of bottom-up molecular design strategies to achieve precise control over the electrical and spin-related properties of 2D c-MOFs.

In anticipation of future advancements in this dynamic field, we envision several promising avenues for further research:

i. Accurate characterization of defects in 2D c-MOFs, including edge terminal defects, and efforts to reduce their density or modify them through chemical strategies are imperative. These endeavors are pivotal for augmenting the stability of the electrical properties of 2D c-MOFs and gaining deeper insights into their intrinsic characteristics, which are foundational for their electronic, spintronic, and quantum applications.
ii. Doping, which encompasses molecular and electrochemical doping, plays a crucial role in fine-tuning organic semiconductors. However, its implementation in 2D c-MOFs remains unexplored. Leveraging the porosity of 2D c-MOFs represents a promising route for accommodating highly dense dopants or counterions, thereby filling the intrinsic defects and achieving an ultrahigh charge carrier concentration. This holds significant potential for augmenting the electrical properties of 2D c-MOFs, particularly in realizing superconductivity.
iii. As potential quantum information carriers, 2D c-MOFs not only require long coherence times but also need a sufficiently large quantum state space as the basis for information processing. Therefore, efforts should be made to achieve a high-spin ground state and construct high-dimensional quantum state spaces of multicenter coupling systems in 2D c-MOFs. Addressing this point may be achieved by using topological engineering to control the coupling and communication of spins within and between 2D layers in 2D c-MOFs, as well as by introducing multiple spin centers in the sample.
iv. Leveraging bottom-up approaches such as molecular design and confined synthesis methods or top-down strategies such as exfoliation to prepare high-quality single-layer or few-layer 2D c-MOFs materials can truly transform bulk 2D c-MOFs into two-dimensional materials. The incoming results will significantly expand the applications of such materials and, more importantly, the intrinsic physics of 2D c-MOFs down to the monolayer level and develop the van der Waals heterostructures based on 2D c-MOFs still largely remain unexplored.

By embracing these future research directions, we anticipate an era of immense possibilities for 2D c-MOFs, expanding their applications in the field of novel quantum materials and making significant contributions to the advancement of this field.
